# Cerebro-Spinal Meningitis—Spotted Fever, &c.

**Published:** 1865-04

**Authors:** N. S. Davis

**Affiliations:** Prof. of Practical and Clinical Medicine


ARTICLE XIV.

### CEREBRO-SPINAL MENINGITIS—SPOTTED FEVER, &c. THE SUMMARY OF A CLINIC IN THE MEDICAL WARDS OF THE MERCY HOSPITAL. APRIL, 1865.

By N. S. DAVIS, M.D., Prof. of Practical and Clinical Medicine.

GENTLEMEN:—The case before you is a boy, aged about 16 years, and until four days since was apparently enjoying good health.

His friends aver that previous to the present attack, he has never had convulsions, epilepsy, or any other nervous or cerebral affection.

His sister states, that, during the early part of last week, he complained of some symptoms of a "common cold," but on Friday evening was suddenly attacked with violent pain in the head; stiffness of the neck; fever, and, in a few hours, general convulsions. The convulsive or spasmodic paroxysm lasted but a few seconds, leaving him, however, entirely unconscious, with rigidity of the muscles of the neck and jaws. Towards morning he had another convulsive paroxysm; after which he remained in the state just described, until he was admitted to the Hospital on Sunday (yesterday) afternoon. The foregoing facts were obtained from a sister of the patient, and are not as complete in relation to the history of the case as is desirable. When admitted to the Hospital, my assistant described his pulse as small and frequent; skin moderately hot; head hot, and face flushed; respiration accelerated, but not noisy; muscles of the neck and jaws rigid, the latter making it very difficult to force the mouth open; deglutition difficult, and the mind apparently unconscious. Cold cloths were applied to his head, and a cathartic of calomel given to move the bowels. The latter having operated early this morning, he was put upon the use of permanganate of potassa, in doses of half a grain dissolved in water, every two hours. At this time (2 o'clock P.M.) he is manifesting some consciousness, and answers questions slowly, but incoherently. The mouth and chin are drawn in a position giving the expression of silliness, or partial dementia, this being much increased when he attempts to speak. The pupils of the eyes are moderately dilated; the skin moderately hot; pulse 115 per minute, and small; respiration easy and quiet; position dorsal, with the muscles of the neck and face slightly rigid. Deglutition is still difficult. There has been some dribbling of urine, and imperfect control over the intestinal evacuations. No petechial or purple spots are visible on the surface. Such being the history and present symptoms of the patient, the important question arises, what is the nature of the disease under which he is suffering? Is it a primary attack of epilepsy, or is it simple inflammatory congestion of the cerebro-spinal centres, or is it a case of that much dreaded and fatal disease, known as cerebro-spinal meningitis, or spotted fever? The case differs from ordinary attacks of epilepsy in several essential particulars. For instance, the first convulsive paroxysm was preceded by severe pain in the head, fever, and stiffness of the neck; while epileptic paroxysms are generally ushered in suddenly without any preceding febrile or inflammatory symptoms. Again the stupor and drowsiness following the epileptic paroxysm is limited to a few minutes or hours, while in this case it has already continued nearly three days, and is only partially relieved. Uncomplicated cases of epileptic convulsions are not followed by either fever or muscular rigidity; both of which are still well-marked in this case. It is evident, then, that the case before us is not one of ordinary epilepsy. That there is a morbid condition of the cerebro-spinal nervous centres, the convulsive movements, the rigidity of the muscles of the neck and face, the difficulty of deglutition, the impaired consciousness, and the imperfect action of the sphincters of the rectum and bladder, clearly indicate. But is this morbid condition one of simple inflammation, or is it accompanied by that peculiar condition of the system which accompanies the epidemic cerebro-spinal meningitis?

The essential pathology of the latter disease, we regard as consisting in a highly septic or pyemic condition of the blood, with perverted vital affinity, accompanied, usually, by local inflammation of the membranes and base of the brain, of such a character as to cause softening and remarkably rapid sero-purulent effusion. The local inflammation in these cases bears the same relation to the morbid condition of the solids and fluids generally, as the local erysipelatous inflammation does to the morbid condition of the blood that precedes and accompanies it, in that form of disease. Like erysipelas, also, the local inflammation is not always developed in the cerebro-spinal centres, but sometimes attacks the lungs, the heart, the serous membranes, &c. Indeed, there is not only a close analogy, but a positive relation between erysipelas and the epidemic cerebro-spinal meningitis or spotted fever; the two diseases having often prevailed together in the same community, and in members of the same family.

There have been some indications of the prevalence of cerebro-spinal meningitis in this city, during the last two or three weeks. At a recent meeting of the Chicago Medical Society, Dr. PERKINS related a case that had just occurred in his practice, in which the symptoms were strongly characteristic, including large numbers of the petechial or purple spots on the skin. The case terminated fatally in a few days. Within the present week I was called to a patient on the North Side of the city, a young man, aged about 25 years, who had been attacked the day previous with a chill, followed by pain in the right side of the chest, and in the head; some cough, which greatly increased the pain; heat of skin; suffusion of the face; pulse 120 per minute, soft and small; respiration accelerated and short. On making a physical examination of the chest, the right side from the clavicle to the diaphragm was less resonant than natural, and over nearly the whole extent of that lung was heard a well-marked sub-crepitant râle. Regarding the case as one of pneumonia, involving nearly the whole of one lung, accompanied by a fever of a typhoid tendency, I directed a powder, containing two grains of sulphate of quinia, two grains of calomel, one-third of a grain of sulphate of morphia, and one grain of pulverized bloodroot, to be taken every four hours, and sinapisms applied extensively to the right side of the chest.

Visiting the patient early the next day, I found him bathed in profuse perspiration; capillaries of the skin congested and leaden color, especially in the face, neck, and extremities; temperature nearly natural; pulse 130 per minute, small and weak; respiration frequent and short, accompanied by abundant mucous rhonchus, with dulness over the right side, the same as the day previous. Several copious, thin, dark-colored evacuations had taken place from the bowels; the expectoration was moderate and slightly mixed with blood; the mind excitedly delirious, and had been constantly so all the preceding night. Although medicines were immediately given that checked the excessive intestinal evacuations, and were calculated to sustain the strength, yet he continued constantly delirious, and every hour more exhausted, until he died on the third day after my first visit. It is true that simple pneumonia, involving the whole of one lung, with the other free, is sufficient to endanger life; but by no means so speedily and with such accompanying symptoms as in this case.

The rapid exhaustion of the patient, accompanied with the constant delirium, and the general capillary congestion, left but little doubt in my mind, that the extensive pulmonary lesion was only coincident with a more general pathological condition of the solids and fluids of the whole system. But no *post mortem* was allowed.

About the same time, another case, almost identical in symptoms, occurred, but under the influence of a powder composed of pulv. gum camphor, five grains; sulphate of quinia, two grains; and pulv. bloodroot, one grain, giving every three hours, aided by the liberal use of chlorate of potassa dissolved in mucilage of gum Arabic, the tendency to exhaustion seemed to be arrested, and the patient slowly recovered.

In the officially compiled table of mortality for the city during the month of March, I notice four deaths attributed to "spotted fever," and two to cerebro-spinal meningitis, while under the vague headings of "spasms" and "convulsions" we have, for the same month, nearly double the number reported during the three preceding months. All these circumstances are sufficient to justify the conclusion that some tendency to cerebro-spinal meningitis actually exists in the city at this time.

That the case now before us partakes of that tendency, the soft, weak pulse; the impaired action of the sphincter muscles; the moderately dilated pupils; and fatuitous expression of countenance, clearly indicate. True, there are no purpuric or hemorrhagic spots. But it is well known that these are often absent in cases of this disease, even when rapidly fatal. We have already indicated our opinion that the epidemic cerebro-spinal meningitis or spotted fever, is a general disease, involving an altered condition both of the blood and the properties of the tissues, accompanied by a special tendency to develop local inflammation in the cerebro-spinal nervous structures of a highly pyemic character. Whether such altered condition of the properties of the solids and fluids of the body is caused by the introduction of some subtle atmospheric poison from without, or by some primary failure in the normal play of those affinities by which nutrition, disintegration and secretion are affected, it is not possible to determine in the present state of medical investigation.

While we may be thus ignorant of the essential or specific cause, the pathological condition itself is abundantly obvious. The practitioner who stands by, watching his patient laboring under a severe attack of this disease, and sees the rapidity with which the nervous, vascular, and secretory functions all fail, as indicated by the muscular rigidity and delirium passing into paralysis and coma; by the universal capillary congestion and frequent appearance of purple spots; by the suspension of important secretions; and by the rapid development of sero-purulent infiltrations and effusions wherever local hyperemia or inflammation exists, will not fail to recognize a profound alteration of the relations or affinities between the ingredients of the blood and the organized structures of the body. Assuming the truth of this position, it is obvious that one of the leading indications in the treatment is to introduce as speedily as possible, into the blood, some medicinal agent which will restore the natural properties or affinities, and thereby arrest the septic or degenerative tendency, the continuance of which so speedily and certainly terminates the life of the patient.

That this cannot be accomplished by any amount of diffusible fully stimulants, anodynes, or evacuants, past experience has demonstrated. It is not a stimulant, soporific, or depletive effect that the patient needs, but rather an antiseptic and alterant influence. The remedies capable of producing the latter effects most efficiently are the chlorine salts, the sulphites, the permanganates, the arsenites, and the cantharides.

The efficacy of the tincture of the chloride of iron in the treatment of erysipelas, is almost universally acknowledged, but we do not recollect seeing the report of any fair trials of it in the treatment of the disease under consideration. In a few cases that came under my care three years since, the sulphites of soda and lime were given freely in conjunction with belladonna, with very satisfactory results. Since then I have not met with a well-marked case of this disease until during the last three months. In the few that have been placed under my care during this latter period, the permanganate of potassa has been used instead of the sulphites; but the number of cases treated has not been sufficient to justify the deduction of any practical conclusions. The results, however, have been such as to justify a much more extended trial of it. The first report concerning its use in the treatment of this disease, that came under my observation, was from a physician of Ohio, whose name I do not recollect, and was very favorable. To afford it a fair trial, its use should be commenced at the earliest moment and continued in such doses as to keep the blood and fluids impregnated with it so long as the severe symptoms continue. At the same time we may allay pain, restlessness, and muscular rigidity, by belladonna, when the pupils of the eyes are contracted, and by morphine when they are larger than natural. Constipation may be obviated by laxatives and stimulating enemas; while cupping, blistering, and other revulsives, may be used to counteract local determinations. In the case before us we will continue the permanganate of potassa in doses of half a grain dissolved in a tablespoonful of cold water, every two hours for the next 24 hours, after which the interval between the doses may be lengthened first to three and subsequently to four hours. We will continue cold applications to the head and warmth to the extremities. As the cathartic given yesterday has operated quite freely, the bowels may show a disposition to become too loose.

If so we will give sufficient emulsion of oil of turpentine and tincture of opium to remedy it. The bladder must also be noted, from time to time, lest retention of urine should take place to an injurious extent.

P.S.—Since writing the foregoing report, the patient, who was the subject of the clinic, has continued gradually to improve, and is now so far recovered as to be out of danger. Certain correspondents will please take the foregoing clinical report as an answer to their letters touching this subject.

